# Intestinal Dysbiosis As a Possible Predictor of Very Early Preterm Labor in Pregnant Women With Metabolic Syndrome

**DOI:** 10.25122/jml-2020-0027

**Published:** 2020

**Authors:** Oksana Mykolaivna Pavlovska, Kateryna Mykolaivna Pavlovska, Svitlana Mykolaivna Heryak, Stefan Volodymyrovych Khmil, Nataliia Yevhenivna Gorban

**Affiliations:** 1. First Department of Obstetrics and Gynecology, Odessa National Medical University, Odessa, Ukraine; 2. Second Department of Obstetrics and Gynecology, I. Horbachevsky Ternopil National Medical University, Ternopil, Ukraine; 3. First Department of Obstetrics and Gynecology, I. Horbachevsky Ternopil National Medical University, Ternopil, Ukraine; 4. Lukianova Institute of Pediatrics, Obstetrics and Gynecology of the NAMS of Ukraine, Kyiv, Ukraine

**Keywords:** Metabolic syndrome, very early preterm labor, intestinal dysbiosis

## Abstract

The work assessed the state of the intestinal microbiocenosis in 52 puerperae at the in whom the pregnancy developed against the background of the metabolic syndrome. The diagnosis of metabolic syndrome was determined according to the criteria approved by the World Health Organization for pregnant women. The state of intestinal microbiocenosis was assessed by a bacteriological examination of feces immediately after delivery. The content of the main representatives of the obligate microflora (bifidobacteria, lactobacilli, native intestinal bacilli, fecal streptococci) and facultative (conditionally pathogenic) microorganisms (representatives of the genus Prоteus, Klebsiella, pathogenic strains of E. coli, Staphylococcus epidermidis, Enterobacter, Citrobacter, Clostridium difficile, Candida fungi) was determined. Cultures were made on appropriate growth media.

At the time of birth, all patients of group I showed signs of intestinal microbiocenosis disorder. At the same time, 13 (54.2%) puerperae were diagnosed signs of dysbiosis of II degree, 9 (37.5%) with signs of III degree, which were generally characterized by a significant decrease in the content of the main representatives of obligate microflora (Bifidobacterium, Lactobacillus, Escherichia coli, Fecal streptococci) with simultaneous high contamination of Candida albicans and Clostridium difficile. So, it can be considered as a possible predictor of very early preterm birth in women with MS. In pregnant women with MS, but who gave timely birth (group II), dysbiotic disorders were detected to a lesser extent. Thus, in 13 (46.4%) patients, initial signs of intestinal dysbiosis (first degree) were detected in 4 (14.3%) patients (second degree). In 11 (39.3%) puerperae of group II, microbial indices indicated normal eubiotic ratios.

## Introduction

According to the World Health Organization (WHO), about 15 million premature births occur annually in the world, and their frequency varies from 5 to 18% [1-3]. According to statistics, more than 80% of preterm births occur during the period of 32–36 weeks of gestation [4-6]. Approximately 60% of preterm birth occurs in the countries of Africa and South Asia. On average, up to 12% of children in developing countries are born premature, and 6–9% in industrialized countries [7-9]. Taking into consideration the fact that Brazil, the USA, India, and Nigeria are among the ten countries with high rates of preterm birth, we can speak about the global nature of this problem [10-13].

This obstetric problem is extremely important in the socioeconomic aspect due to the high mortality rate, various severe perinatal pathologies, and the risk of future disability. Thus, prematurity ranks second among the leading causes of death among children under the age of 5. The results of multi-center studies have shown an extremely high risk for preterm infant encephalopathy due to hyperbilirubinemia and hypoglycemia, intraventricular hemorrhage, pneumonia, respiratory distress syndrome, necrotizing enterocolitis, sepsis, retinopathy (high myopia, hypermetropia of a high degree, blindness), hearing loss up to deafness [14-20]. In the more distant future, premature infants often develop motor impairments, including spastic cerebral palsy, cognitive impairments, dyslexia, poor performance, attention deficit, elevated levels of anxiety or depression are also characteristic [21-23].

It should be noted that certain knowledge has been accumulated about the etiological factors and pathogenetic mechanisms of preterm labor at the present stage of development of obstetrics. Clinical protocols for the management of pregnant women with this pathology at the outpatient and inpatient stages are being developed, implemented, and constantly improved.

One of the significant etiopathogenetic factors of preterm labor is currently recognized as metabolic syndrome (MS) [24-26]. According to modern concepts, MS is a complex of metabolic and hormonal disorders that are accompanied by insulin resistance/compensatory hyperinsulinism, and cause, first of all, systemic dysregulation of the vascular tone and endothelial dysfunction [27-30].

It should be emphasized that some researchers associate the development and progression of MS with intestinal dysbiosis [31]. It is known that the intestinal microflora is actively involved in the regulation of metabolic homeostasis in the body, exerting a direct influence on the activity of vegetative, neuroendocrine [32], and immune interactions in the macroorganism [33]. It has been established that during pregnancy occurring against the background of MS, premature uterine contractile activity inducing abortion may occur due to the imbalance between vasoconstriction/vasodilation factors, inflammatory mediators/ prostaglandins, and steroid hormones [34].

In our investigation, we studied the characteristics of the intestinal microbiocenosis in women suffering from MS and giving birth within 22-27 weeks (very early preterm labor). In our opinion, the information obtained will allow to a certain extent to clarify and supplement the existing clinical algorithms in the management of pregnant women with extragenital pathologies and the risk of preterm birth and the objective was to study the state of intestinal microbiota in women with MS who have had very early preterm labor.

## Material and Methods

A clinical and laboratory examination of 52 pregnant women with MS at the age of 24-42 years old who were divided into 2 groups was conducted.

• Group I (n = 24) included pregnant women with MS who had a gestational birth at 22-27 weeks (very early preterm birth).
• Group II (n = 28) included women with MS who had timely delivery.

The control group consisted of 25 healthy women, in whom the pregnancy took a normal course without any complications, and the delivery took place in time.

The diagnosis of MS was determined according to the criteria approved by the WHO for pregnant women:

1. Insulin resistance (type 2 diabetes, fasting glucose ≥5.8 mmol/l);
2. The presence of two or more additional features (blood pressure >140/90 mm Hg; triglyceride level ≥ 1.7 mmol/l; the level of high-density lipoprotein ≤1.1 mmol/l; body mass index> 30 kg/m^2^).

The diagnosis of type 2 diabetes was established on the basis of the criteria approved by the WHO (2-fold fasting glucose ≥7.0 mmol/l (126 mg/dL); glycemia ≥11.1 mmol/l two hours after the oral glucose tolerance test (200 mg/dL); accidental glycemia ≥11.1 mmol/l (200 mg/dL) and the accompanying symptoms of hyperglycemia (sharp thirst, pruritus, frequent urination, irritability, fatigue, loss of consciousness and coma in severe cases).

The state of intestinal microbiocenosis was assessed by a bacteriological examination of feces immediately after delivery. The content of the main representatives of the obligate microflora (bifidobacteria, lactobacilli, native intestinal bacilli, fecal streptococci) and facultative (conditionally pathogenic) microorganisms (representatives of the genus Prоteus, Klebsiella, pathogenic strains of E. coli, Staphylococcus epidermidis, Candida fungi) was determined. Cultures were made on appropriate growth media. Quantitative calculation of the grown microorganisms on yolk-salt agar, Saburo, Endo and 5% blood agar was carried out by calculating 1 g of feces, taking into account the dose of the cultured material and the degree of its dilution. The evidence of dysbiotic disorders was assessed by the degree:

• First-degree of microbiological disorders - a decrease in the content of Bifidobacterium up to 108 - 107 CFU/g, Lactobacillus - up to 106 - 105 CFU/g, Escherichia coli - up to 106 - 105 CFU/g; there may be an increase in the content of Escherichia coli up to 109 - 1010 CFU/g.
• Second-degree of microbiological disorders – reduction of the content of Bifidobacterium up to 107 CFU/g and below, Lactobacillus – up to 105 CFU/g and below, increase of the content of hemolytic Escherichia or other conditionally pathogenic bacteria to a concentration of 105-107 CFU/g or detection of associations of conditionally pathogenic microorganisms in the concentration of 104 -103 CFU/g.
• Third-degree of microbiological disorders - reduction of the content of Bifidobacterium up to 107 CFU/g and below, Lactobacillus - up to 105 CFU/g and below, detection of associations of conditionally pathogenic microorganisms in a concentration of 106 - 107 CFU/g and higher.

For processing the results of the study, the method of variation statistics, as well as the non-parametric methods were performed using Excel 2000 and Statistica for Windows v.6.0.

## Results and Discussion

In the statistical processing of the research results, very early preterm labor in women of group I occurred at 24.5 ± 0.24 weeks. The patients in group II gave birth at 38.1 ± 0.17 weeks, and women in the control group at 39.1 ± 0.19 weeks.

It should be noted that in women of the first group, pregnancy had an unfavorable course from the early terms of gestation (Table 1). Thus, 19 patients (79.2%) received treatment for threatened miscarriage in the first trimester. Pregnancy vomiting was diagnosed in 13 (54.2%) cases, not severe preeclampsia in 5 (20.8%) cases, severe preeclampsia in 2 (8.3%) cases, premature detachment of the normally located placenta in 3 (12.5%) cases, placental dysfunction in 23 (95.8%) cases, signs of delayed fetal development were detected in 22 (91.7%) cases, fetal distress in 1 (4.2%) case, premature rupture of the amniotic fluid in 2 (8.3%) cases.

**Table 1:** The frequency of obstetric complications in patients with MS who had very early preterm labor (group I) and who gave birth in time (group II).

**Obstetric complications**	**Group I,** **n=24**		**Group II,** **n=28**	
	**abs.**	**%**	**abs.**	**%**
**Threatened miscarriage**	19	79.2	11	39.3
**Vomiting of pregnant women**	13	54.2	15	53.6
**Not severe preeclampsia **	5	20.8	3	10.7
**Severe preeclampsia**	2	8.3	2	7.1
**Premature detachment of a normally located placenta**	3	12.5	1	3.6
**Premature rupture of the amniotic fluid**	2	8.3	2	7.1
**Placental dysfunction**	23	95.8	17	60.7
**Fetal distress**	1	4.2	2	7.1
**Fetal growth retardation**	22	91.7	17	60.7

Considering the high level of obstetric complications in the patients of this group, we analyzed the concomitant somatic diseases (Table 2). It was revealed that the most frequent comorbidities in the women under investigation were diseases of the cardiovascular system and inflammatory diseases of the internal organs.

**Table 2:** Concomitant somatic diseases in patients with MS who had very early preterm labor (group I) and those delivered in time (group II).

**Somatic pathology**	**Group I,** **n=24**		**Group II,** **n=28**	
	**abs.**	**%**	**abs.**	**%**
**Chronic hypertension**	7	29.2	6	21.4
**Nodular goiter of I-II degree**	2	8.3	-	-
**Anemia**	6	25.0	8	28.6
**Chronic venous insufficiency**	3	12.5	3	10.7
**Chronic bronchitis**	1	4.2	-	-
**Chronic tonsillitis**	4	16.7	2	7.1
**Bronchial asthma**	1	4.2	-	-
**Chronic pyelonephritis**	5	20.8	2	7.1
**Chronic cystitis**	2	8.3	-	-
**Chronic adnexitis**	7	29.2	4	14.3
**Hormonal ovarian dysfunction**	5	20.8	6	21.4

In women of group II, pregnancy was also complicated, but the frequency of obstetric complications was somewhat lower. Thus, signs of threatened miscarriage in the first trimester were detected in 11 (39.3%) patients of this group, i.e., almost two times less compared with group I. Vomiting was diagnosed in 15 (53.6%) pregnant women, not severe preeclampsia in 3 (10.7%), severe preeclampsia in 2 (7.1%), fetal development retardation in 17 (60.7%), fetal distress in 2 (7.1% ), premature detachment of the normally located placenta in 1 (3.6%), placental dysfunction in 17 (60.7%), premature rupture of the amniotic fluid in 2 (7.1%).

In the majority of the patients of groups I and II, there was vaginal delivery in 18 (75.0%) and 23 (82.1%) women, respectively. Surgical delivery by cesarean section was performed in 6 (25.0%) and 5 (17.9%) pregnant women of groups I and II, respectively.

The next step was to identify the features of the intestinal microbiocenosis in the patients suffering from MS and a comparison of the results obtained in the control group.

Thus, a comparative analysis of the results of a bacteriological examination of feces in the patients with MS who gave birth prematurely (group I) revealed significant disorders of the intestinal microecology compared with the patients of group II and healthy puerperae (Tables 3, 4).

**Table 3:** Intestinal dysbiosis at the time of delivery in patients with MS (groups I and II) and healthy patients (control group).

**A degree of intestinal dysbiosis**	**Group I,** **n=24**		**Group II,** **n=28**		**Control group,** **n=25**	
	**abs.**	**%**	**abs.**	**%**	**abs.**	**%**
**No signs of dysbiosis**	-	-	11	39.3	18	72.0
**Intestinal dysbiosis of first-degree**	2	8.3	13	46.4	4	16.0
**Intestinal dysbiosis of second-degree**	13	54.2	4	14.3	3	12.0
**Intestinal dysbiosis of third-degree**	9	37.5	-	-	-	-

**Table 4:** Indices of the content of microorganisms in 1g of feces at the time of delivery in patients with MS (group I, II) and healthy patients (control group), M ± m.

**Microorganisms**	**Group I,** **n=24**	**Group II,** **n=28**	**Control group,** **n=25**
**Lactobacillus (×10**6**)**	0.67±0.15 (pI-II<0.001) (pI-К<0.001)	6.42±0.71 (pII-К=0.027)	18.56±5.28
**Bifidobacterium (×10**8**)**	1.31±0.62 (pI-II<0.001) (pI-К<0.001)	9.68±2.16 (pII-К=0.008)	27.58±6.15
**Fecal streptococci (×10**6**)**	0.32±0.10 (pI-II=0.069) (pI-К=0.026)	0.52±0.04 (pII-К=0.326)	0.60±0.07
**Escherichia coli (×10**6**)**	5.82±1.20 (pI-II<0.001) (pI-К<0.001)	30.52±7.40 (pII-К=0.011)	60.54±8.54
**Enterococcus faecium (×10**6**)**	7.02±1.18 (pI-II<0.001) (pI-К<0.001)	28.12±5.33 (pII-К=0.017)	54.36±9.24
**Staphylococcus epidermidis (×10**4**)**	3.14±0.60 (pI-II=0.006) (pI-К<0.001)	1.30±0.24 (pII-К<0.001)	0.31±0.08
**Enterobacter (×10**3**)**	4.52±1.28 (pI-II=0.033) (pI-К=0.014)	1.41±0.60 (pII-К=0.772)	1.23±0.15
**Citrobacter (×10**3**)**	3.19±1.21 (pI-II=0.286) (pI-К=0.169)	1.79±0.47 (pII-К=0.541)	1.48±0.18
**Proteus (×10**3**)**	5.10±1.18 (pI-II=0.020) (pI-К=0.002)	2.01±0.51 (pII-К=0.104)	1.03±0.30
**Klebsiella (×10**5**)**	2.14±1.03 (pI-II=0.090) (pI-К=0.084)	0.36±0.03 (pII-К=0.427)	0.32±0.04
**Clostridium difficile (×10**5**)**	2.54±0.63 (pI-II <0.001) (pI-К <0.001)	0.28±0.04 (pII-К=0.168)	0.21±0.03
**Candida albicans (×10**4**)**	3.90±0.63 (pI-II<0.001) (pI-К<0.001)	0.37±0.03 (pII-К=0.006)	0.15±0.07

Note: pI-II – reliable differences between indices of group I and group II;

pI-K – reliable differences between indices of the first group and the control group;

pII-K – reliable differences between the indices of the second group and the control group.

At the time of birth, all patients of group I showed signs of intestinal microbiocenosis disorder. At the same time, 13 (54.2%) puerperae were diagnosed with signs of dysbiosis of second-degree, and 9 (37.5%) with third-degree dysbiosis. In pregnant women with MS, but who gave timely birth (group II), dysbiotic disorders were detected to a lesser extent. Thus, in 13 (46.4%) and 4 (14.3%) patients, there were initial signs of intestinal dysbiosis of first-degree) and second-degree, respectively. In 11 (39.3%) puerperae of group II, microbial indices indicated normal eubiotic ratios.

It should be emphasized that in the patients of group I who had very early preterm labor, there was a significant inhibition of the growth of the main representatives of obligate microflora, which was accompanied by excessive reproduction of various types of conditionally pathogenic microorganisms. The content of Bifidobacterium was determined at the level of (1.31 ± 0.62) ∙ 108 (pI-II <0.001, pI-K <0.001), Lactobacillus - (0.67 ± 0.15) ∙ 106 (pI-II <0.001, pI-K <0.001), Escherichia coli - (5.82 ± 1.20) ∙ 106 (pI-II <0.001, pI-K <0.001), Enterococcus faecium - (7.02 ± 1.18) ∙ 106 (pI-II <0.001, pI-K <0.001). The content of Fecal streptococci in feces was (0.32 ± 0.10) ∙ 106 (pI-II = 0.069, pI-K = 0.026), Klebsiella - (2.14 ± 1.03) ∙ 105 (pI-II = 0.090, pI-K = 0.084) Citrobacter - (3.19 ± 1.21) ∙ 103 (pI-II = 0.286, pI-K = 0.169), which is not statistically significantly different from those of the patients in group II and the control group.

However, the levels of Staphylococcus epidermidis, Proteus, Enterobacter, Clostridium difficile and Candida albicans were several times higher than the compared indices and were within (3.14 ± 0.60) ∙ 104 (pI-II = 0.006, pI-K <0.001), (5.10 ± 1.18) ∙ 103 (pI-II = 0.020, pI-K = 0.002), (4.52 ± 1.28) ∙ 103 (pI-II = 0.033, pI-K = 0.014), (2.54 ± 0.63) ∙ 105 (pI-II <0.001, pI-K <0.001) and (3.90 ± 0.63) ∙ 104 (pI-II <0.001, pI-K <0.001), respectively.

In puerperae of group II, a statistically significant decrease in the content of Bifidobacterium up to (9.68 ± 2.16) ∙ 108 (pII-K = 0.008), Lactobacillus – up to (6.42 ± 0.71) ∙ 106 (pII-K = 0.027), Escherichia coli – up to ( 30.52 ± 7.40) ∙ 106 (pII-K = 0.011), Enterococcus faecium - (28.12 ± 5.33) ∙ 106 (pII-K = 0.017) compared with the control group, but less pronounced compared to group I. High contamination with Candida albicans - (0.37 ± 0.03) ∙ 104 (рII-K = 0.006) and Staphylococcus epidermidis - (1.30 ± 0.24) ∙104 (рII-K <0.001) was also detected. Indices of conditionally pathogenic bacteria such as Fecal streptococci, Proteus, Enterobacter, Citrobacter, Clostridium difficile and Klebsiella did not statistically differ from the control values - (0.52 ± 0.004) ∙ 106 (pII-K = 0.326), (2.01 ± 0.51) ∙ 103 (pII-K = 0.104), (1.41 ± 0.60) ∙ 103 (pII-K = 0.772), (1.79 ± 0.47) ∙ 103 (pII-K = 0.541), (0.28 ± 0.04) ∙ 105 (pII-K = 0.168) and (0.36 ± 0.03) ∙ 105 (pII-K = 0.427), respectively.

The obtained results suggest that there are significant dysbiotic disorders in the intestine regarding very early preterm labor in women with MS. Intestinal dysbiosis is accompanied by a decrease in the main representatives of the indigenous microflora (Bifidobacterium, Lactobacillus, Escherichia coli, Fecal streptococci) with high contamination of Clostridium difficile and Candida albicans and, in pregnant women with MS, this could be one of the mechanisms of initiation of premature termination of pregnancy, or an aggravating factor that requires further in-depth study and analysis.

## Conclusions

In the group of patients with MS who had very early preterm labor, a marked imbalance of the intestinal microbiota was registered.

The overwhelming majority (91.7%) of the women who had very preterm labor were diagnosed with intestinal microecology disorders of the second and third-degree. The intestinal microbial landscape was characterized by a significant decrease in the content of the main representatives of obligate microflora (Bifidobacterium, Lactobacillus, Escherichia coli, Fecal streptococci) and increased growth of conditionally pathogenic bacteria (Staphylococcus epidermidis, Proteus, Enterobacter, Clostridium difficile and Candida albicans). In pregnant women with MS, but who gave timely birth, dysbiotic disorders were detected to a lesser extent.

It should be emphasized that intestinal dysbiosis, accompanied by a significant decrease in the indigenous microflora with simultaneous high contamination of Candida albicans and Clostridium difficile, can be considered as a possible predictor of very early preterm birth in women with MS. Through MS correction and timely therapeutic modulation of the intestinal microbiota (dietary, medication) is quite possible to reduce the incidence of premature birth in pregnant women with this pathology; therefore, this is a promising direction for future research.

## Conflict of Interest

The authors confirm that there are no conflicts of interest.
